# Interplay between small and long non‐coding RNAs in cutaneous melanoma: a complex jigsaw puzzle with missing pieces

**DOI:** 10.1002/1878-0261.12412

**Published:** 2018-12-20

**Authors:** Mattia Riefolo, Elisa Porcellini, Emi Dika, Elisabetta Broseghini, Manuela Ferracin

**Affiliations:** ^1^ Department of Experimental, Diagnostic and Specialty Medicine (DIMES) University of Bologna Italy

**Keywords:** cutaneous melanoma, microRNA, non‐coding RNA

## Abstract

The incidence of cutaneous melanoma (CM) has increased in the past few decades. The biology of melanoma is characterized by a complex interaction between genetic, environmental and phenotypic factors. A greater understanding of the molecular mechanisms that promote melanoma cell growth and dissemination is crucial to improve diagnosis, prognostication, and treatment of CM. Both small and long non‐coding RNAs (lncRNAs) have been identified to play a role in melanoma biology; microRNA and lncRNA expression is altered in transformed melanocytes and this in turn has functional effects on cell proliferation, apoptosis, invasion, metastasis, and immune response. Moreover, specific dysregulated ncRNAs were shown to have a diagnostic or prognostic role in melanoma and to drive the establishment of drug resistance. Here, we review the current literature on small and lncRNAs with a role in melanoma, with the aim of putting into some order this complex jigsaw puzzle.

**Abbreviations**
ADAR: double‐stranded RNA‐specific adenosine deaminase
AID: activation‐induced cytidine deaminase
AKT: activity of protein kinase B
ANRIL: antisense non‐coding RNA in the INK4A locus
BANCR: BRAF‐activated non‐coding RNA
circRNA: circular RNA
CM: cutaneous melanoma
CSD: chronically sun‐damaged
CTL: cytotoxic T lymphocytes
EGR2: early growth response 2
EMT: epithelial‐mesenchymal transition
EVs: extracellular vesicles
FPGS: folylpolyglutamate synthase
GPR 55: G‐protein‐coupled receptor 55
IL: interleukin
ITGB1: integrin β1
lncRNA: long non‐coding RNA
miRNA: microRNA
MITF: microphthalmia‐associated transcriptional factor
mRNA: messanger RNA
ncRNA: non‐coding RNA
NF‐κB1: nuclear factor kappa‐light‐chain‐enhancer of activated B cells
NK: natural killer
NPL1: neuropilin‐1
oncomiR: oncogenic microRNA
PDX: patient‐derived xenograft
PRC: polycomb repressor complex
PVT1: plasmacytoma variant translocation 1
Rb: retinoblastoma protein
SOX2: transcription factor SOX‐2
TCGA: The Cancer Genome Atlas
TGFβ: transforming growth factor beta
TIMP3: tissue inhibitor of metalloproteinase‐3
TNF: tumor necrosis factor

## 1 Clinical aspects of cutaneous melanoma

The incidence of cutaneous melanoma (CM) has increased in the past few decades. The worldwide highest incidence is reported in Australia (36–50/100 000 people). In Europe, the lowest incidence occurs in the Southern and the highest in the Northern countries (10–20 cases/100 000) (Arnold *et al*., 2014). CM accounts for 3–5% of all cutaneous cancers. Most cases of CM are diagnosed at an early stage and are curable with surgical excision. On the other hand, the diagnosis of an advanced CM represents a therapeutic challenge, due to the low sensitivity to chemotherapy demonstrated by this tumor (Viros *et al*., 2008).

The 5‐year survival for CM is 91% overall. Patients with early‐stage disease have a 5‐year survival rate of about 98%, but this rate drastically decreases when regional or distant metastases are present, ranging from 63% to 16%, respectively (Box and Terzian, 2008; Haferkamp *et al*., 2009; McKenzie *et al*., 2010).

The pathogenesis of CM is complex and includes genetic, environmental (UV radiation exposure) and phenotypic factors (fair phototypes, multiple nevi, positive family history for melanoma). Studies on germline mutations focusing on the cyclin‐dependent kinase inhibitor 2A (*CDKN2A*), have shown mutations in 5–15% of familial cases affected by CM. Other susceptibility genes include *MITF, CDK4, POT1, ACD, TERF2IP, BAP1* and *TERT* promoter (van Dijk *et al*., 2005; Garrido and Bastian, 2010; Hacker *et al*., 2010; Jovanovic *et al*., 2010; Soura *et al*., 2016). Nevertheless, the majority of melanomas are non‐familial and sporadic. For these patients, the recognition of the orchestra of genetic and epigenetic regulatory mechanisms involved in the development and progression of melanoma has permitted in the last two decades an improvement in the clinical management of patients affected by metastatic disease. Indeed, the knowledge of genetic alterations in primary and metastatic tumors has offered clinically actionable targets.

**Figure 1:**
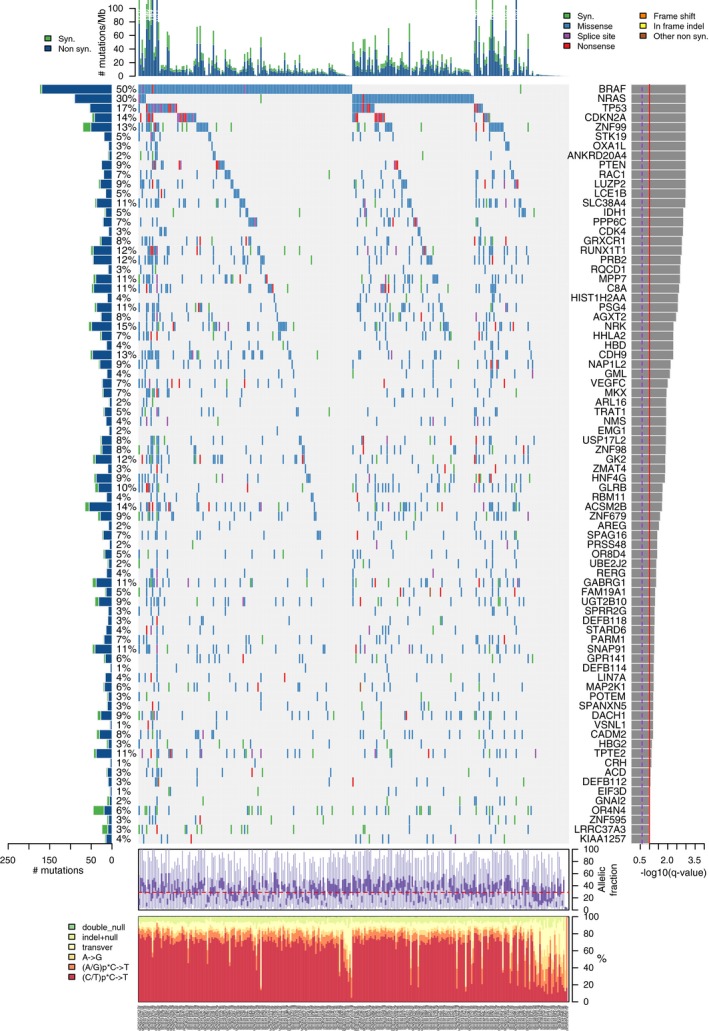
Plot of significantly mutated genes from the mutsig2.0 computational analysis of whole‐exome sequencing data from a SKCM TCGA cohort obtained from the Firebrowse website (Cancer Genome Atlas Research, 2011). Genes are ordered by level of significance (*q* value at right). At left is the prevalence of each mutation in the sample set. The top graph shows the number of mutations per sample, subclassified as synonymous (Syn) and non‐synonymous (Non syn). The bottom plots show the distribution of allelic fraction of mutations for each sample and the frequency of the different types of genetic alterations.

Cutaneous melanoma has one of the highest genomic mutational burdens (number of mutations per megabase) among human cancers (Chalmers *et al*., 2017). This is specifically true for the cutaneous subtype and not acral or mucosal subtypes, because of the effect of UV exposure. Recently, the landscape of genomic alterations characterizing human cancers has been made available through The Cancer Genome Atlas (TCGA) project. In the context of this project, 333 samples of primary and metastatic cutaneous melanoma (SKCM) were analyzed by Whole Exome Sequencing and classified into four main genomic subtypes: mutant *BRAF*, mutant *NRAS*, mutant neurofibromatosis type 1 (*NF1*) and triple‐wild‐type (Cancer Genome Atlas, 2015). In 2017, a more detailed analysis of whole genome alterations of 183 melanoma samples reported *BRAF, CDNK2A, NRAS* and *TP53* as the most frequently mutated genes in CM (Hayward *et al*., 2017). When copy number variations were included in this classification, CM was characterized by dysregulation in the following signaling pathways: MAPK in 92% of the samples, PI3K in 56%, RTKs in 48%, Histone modification in 48%, Cell cycle in 40%, SWI/SNF in 38%, TP53 in 37% and WNT in 29%. In Fig. 1, we present a picture of the most significantly mutated genes in melanoma, obtained using TCGA SKCM samples.

MAPK pathway members include RAS, RAF, MEK and ERK. The main function of the MAPK pathway is to transfer extracellular signals from the cell membrane to the nucleus, using protein phosphorylation, finally promoting cell proliferation (van Dijk *et al*., 2005; Jovanovic *et al*., 2010; Mishra *et al*., 2010; Takata and Saida, 2006). Understanding of this pathway is hindered by the presence of multiple isoforms of the RAS, RAF, MEK and ERK proteins, which have different functions and whose specific regulatory aspects are yet not fully understood.

The RAF family consists of A‐RAF, BRAF and C‐RAF, which are protein kinases frequently mutated in cancer. *BRAF* gene mutations are found in 52% of melanomas (Cancer Genome Atlas Network, 2015), and 90% of those mutations are a single nucleotide alteration (nucleotide 1799 T>A), resulting in substitution of glutamic acid for valine (BRAFV600E) (Ascierto *et al*., 2012). This mutation causes the constitutive activation of the kinase and also insensitivity to negative feedback mechanisms, finally promoting angiogenesis (via HIFα and VEGF activation), apoptosis evasion, invasion and metastasis (Maurer *et al*., 2011). BRAFV600E also regulates interleukin (IL)‐8, which promotes the adhesion of melanocytes to the vasculature, thereby helping to promote metastases (Singh *et al*., 1994). In addition, *BRAF* mutations have been also detected in typical and atypical melanocytic nevi. In nevi, *BRAF* mutations initially trigger growth in lesions that will eventually stop proliferating and remain benign (Michaloglou *et al*., 2005). This oncogene‐induced senescence is theorized to occur in the 82% of melanocytic nevi with *BRAF* mutations (Wajapeyee *et al*., 2008). A second mutation causing the loss of a tumor‐suppressor gene or causing a second mutation could cause a transition from ‘benign’ *BRAF*‐mutated nevi to malignant melanoma (Arkenau *et al*., 2011; Jovanovic *et al*., 2010; McKenzie *et al*., 2010; Michaloglou *et al*., 2005).

The RAS family of proteins contains N‐RAS, K‐RAS and H‐RAS. NRAS mutations have been found in 28% of melanomas (Cancer Genome Atlas Network, 2015), mostly occurring on chronically sun‐exposed skin (van Dijk *et al*., 2005; Dong *et al*., 2003; Jovanovic *et al*., 2010). Most mutations in *NRAS* occur at codon 61 and make the protein constitutively active. Mouse models have revealed a requirement for concurrent loss of *CDKN2A/p16*, a tumor‐suppressor gene, to develop melanoma (Dong *et al*., 2003). *NRAS* mutations are mutually exclusive with *BRAF* mutations with very rare exceptions (van Dijk *et al*., 2005). *KRAS* and *HRAS* mutations have been found respectively in 2% and 1% of CM.

MEK1 and 2 are protein kinases that are downstream of BRAF. MEK is active in 30% of all cancers. Inhibitors for the protein have been developed as therapeutic targets for BRAF‐mutated melanomas (McKenzie *et al*., 2010).

ERK1 and 2 are the only proteins downstream of MEK. They phosphorylate microphthalmia‐associated transcriptional factor (MITF), which regulates melanocyte differentiation. ERK activation has been reported in melanoma, and activated ERKs are present at lower levels in the melanocytes of normal‐appearing skin.

In the last few years, the detection of somatic mutations, the development of target therapies, and the introduction of immune checkpoint inhibitors have led to important therapeutic advances for melanoma patients (Kirchberger *et al*., 2018).

The first targeted therapy to be introduced was monotherapy with BRAF inhibitors such as vemurafenib or dabrafenib for the treatment of advanced BRAF‐mutant melanoma (Martin‐Liberal and Larkin, 2015). These drugs improve the outcome of patients with advanced BRAF V600‐mutant melanoma, with a high rate of tumor response and improvement in progression‐free survival and overall survival compared with standard cytotoxic chemotherapy. Nonetheless, the acquired resistance to BRAF inhibitor monotherapy represents the most common cause of treatment failure, due to the reactivation of MAPK pathway through MEK.

MEK inhibitors were than introduced in combination with BRAF‐targeted therapy, demonstrating benefits in randomized clinical trials, not solely attenuating the development of resistance but also improving progression‐free survival and overall survival with respect to BRAF inhibitors alone (Long *et al*., 2014; Mai *et al*., 2015; Spagnolo *et al*., 2014).

Recently, immunotherapy with checkpoint inhibitors targeting cytotoxic T lymphocyte‐associated antigen 4, CTLA‐4 (ipilimumab) and programmed death 1, PD‐1 (pembrolizumab and nivolumab) have also been demonstrated to provide durable effects in metastatic melanoma, which improved when combined together (Larkin *et al*., 2015; Weber *et al*., 2017; Wolchok *et al*., 2017).

Future investigations are needed to better comprehend the mechanism of primary or secondary resistance to immune checkpoint inhibitors and targeted therapies. This could be avoided by targeting other players in the tumor microenvironment or acting on other dysregulated molecules inside the tumor cell, including non‐coding RNAs (ncRNAs).

## 2 Non‐coding RNA dysregulation in cancer

In the past two decades, dysregulated expression of small ncRNAs, including microRNAs (miRNAs), and lncRNAs has been reported in many, if not all, tumor types. The relevance of ncRNA in cancer development is becoming clearer with every passing day: ncRNAs constitute an additional layer of complexity in the cellular regulatory machinery and their alteration is a well‐established driver of cancer in addition to genetic, epigenetic and protein‐coding gene expression dysregulation. Much effort has focused on identifying which ncRNA molecules are altered in each cancer type, including melanoma.

The ENCODE project revealed that 80% of the human genome is biochemically functional (Djebali *et al*., 2012). Non‐coding transcripts constitute the majority of RNA molecules generated from the active genome, making up 97% of the transcriptome (Moraes and Goes, 2016). The exact type, role and function of all non‐coding transcripts is still under evaluation, although some ncRNA classes have been better studied than others. Among the small ncRNAs (size < 200 nt), the most studied are undoubtedly miRNAs. It is well known that human miRNAs can actively regulate protein‐coding gene transcription by binding to the 3′‐UTR of target mRNAs, therefore inducing their degradation or blocking their transcription. We also know that lncRNAs fulfil a series of regulatory functions in the cell, including organization of nuclear architecture, recruitment of chromatin‐modifying proteins, modulation of protein–DNA binding, and regulation of mRNA stability and translation (Paralkar and Weiss, 2013; Rutenberg‐Schoenberg *et al*., 2016; Tan and Marques, 2014; Tan *et al*., 2014; Vance and Ponting, 2014).

In 2012, thousands of circular RNAs (circRNAs) were discovered (Salzman *et al*., 2012). circular RNAs are particularly stable and resistant to RNA degradation and act as very powerful ‘miRNA sponges’. According to the competing endogenous RNAs hypothesis, any RNA molecule can potentially regulate the level of multiple transcripts by subtracting miRNAs from other RNAs that share the same miRNA responsive element (Salmena *et al*., 2011). Starting from this scenario, we can only begin to imagine how complex the interactions between coding and non‐coding RNAs could be. Indeed, dysregulated lncRNAs could indirectly modulate mRNA levels by competing for miRNA targeting the same gene, thus altering protein localization and function.

In this review, we cover the state‐of‐the‐art research on ncRNAs in melanoma by presenting and discussing all the most relevant studies published from 2008 to 2018.

### 2.1 MicroRNA dysregulation in melanoma

Since the discovery of miRNAs, several studies have reported miRNA dysregulation in human cancers, including melanoma (Hanniford *et al*., 2015; Howell *et al*., 2010; Kozubek *et al*., 2013; Mueller *et al*., 2009; Philippidou *et al*., 2010; Stark *et al*., 2010). Depending on the targets they regulate and the tissue where they are expressed, miRNAs can have oncogenic or tumor‐suppressive roles. Oncogenic miRNAs, known as oncomiRs, target and downregulate tumor‐suppressor genes. On the other hand, some miRNAs can have a protective role downregulating genes associated with growth and metastasis. An imbalance of these two types of miRNA, specifically upregulation of oncomiRs and downregulation of tumor‐suppressor miRNAs, affects tumor development and progression (Negrini *et al*., 2007; Zhang *et al*., 2007).

For a better understanding of the functional role of miRNAs in melanoma, we organized the main experimental discoveries according to the cancer hallmark that is influenced by the miRNA. These results are summarized in Table 1.

**Table 1:** MicroRNAs (miRNAs) dysregulated in human cutaneous melanoma and classified according to their main function in tumor cells

miRNA^a^	Expression in melanoma vs. normal melanocytes	Target gene(s)	Reference(s) on target gene regulation
Melanoma biology			
211‐5p	Downregulated	MITF/TRPM1	Hammock *et al*. (2006)
	Downregulated	NUAK1	Bell *et al*. (2014)
	Downregulated	ERK	Vitiello *et al*. (2017)
182‐5p, 137	Upregulated, not‐specified	MITF	Bemis *et al*. (2008), Segura *et al*. (2009)
Cell proliferation and cycle			
21‐5p	Upregulated	PDCD4, PTEN, BTG2	Yang *et al*. (2011)
155‐5p	Upregulated	SKI	Levati *et al*. (2011)
135a‐5p	Upregulated	FOXO1	Ren *et al*. (2015)
145‐5p	Downregulated	c‐MYC	Noguchi *et al*. (2012a)
125b‐5p	Downregulated		Nyholm *et al*. (2014)
Let‐7 family	Downregulated	Rb	Schultz *et al*. (2008)
206	Downregulated	CDK4, CCND1, Cyclin C	Georgantas *et al*. (2014)
193b‐3p	Downregulated	CCND1	Chen *et al*. (2010)
137	Downregulated	c‐Met, YB1, MITF, EZH2	Luo *et al*. (2013b)
365a‐3p	Downregulated	CCND1	Zhu *et al*. (2018)
101‐3p	Downregulated	MITF, EZH2	Luo *et al*. (2013a)
205‐5p	Downregulated	ZEB2	Liu *et al*. (2012b)
	Downregulated	E2F1, E2F5	Dar *et al*. (2011)
203a‐3p	Downregulated	BMI1	Chang *et al*. (2015)
	Downregulated	E2F3a, E2F3b	Noguchi *et al*. (2012b)
126‐3p, 126‐5p	Downregulated	ADAM9, MMP7	Felli *et al*. (2013)
194‐5p	Downregulated	PI3K/AKT/FoxO3a	Bai *et al*. (2017)
	Downregulated	GEF‐H1	Guo *et al*. (2016b)
485‐5p	Downregulated	FZD7	Wu *et al*. (2017)
136‐5p	Downregulated	PMEL	Wang *et al*. (2017b)
31‐5p	Downregulated	PI3K/AKT	Zheng *et al*. (2018)
Apoptosis			
18b‐5p	Downregulated	MDM2	Dar *et al*. (2013)
638	Upregulated	TP53INP2	Bhattacharya *et al*. (2015)
21‐5p	Upregulated	PDCD4, PTEN, BTG2	Yang *et al*. (2011)
	Upregulated	PTEN, BCL‐2, pAKT	Syed *et al*. (2013)
4286	Upregulated	FPGS, RRN3, APLN, GPR 55, HMGA1	Komina *et al*. (2016)
15b‐5p	Upregulated	Caspase 3 and 7 and Annexin V	Satzger *et al*. (2010)
125b‐5p	Downregulated		Glud *et al*. (2010), Holst *et al*. (2011), Nyholm *et al*. (2014)
205‐5p	Downregulated	E2F1, RB	Dar *et al*. (2011)
26a‐5p	Downregulated	SODD	Reuland *et al*. (2013)
Invasion and metastasis			
150‐5p	Upregulated	MYB, EGR2, NOTCH3, cytokine signaling cascade	Fleming *et al*. (2015), Howard *et al*. (2013), Kunz (2013)
211‐5p	Downregulated	TGFB	Levy *et al*. (2010)
	Downregulated	BRN2	Boyle *et al*. (2011)
	Downregulated	KCNMA1	Mazar *et al*. (2010)
101‐5p	Downregulated	MITF, EXH2	Luo *et al*. (2013a)
200c‐3p	Upregulated	MARCKS	Elson‐Schwab *et al*. (2010)
203a‐3p	Downregulation	BMI1	Chang *et al*. (2015)
9‐5p	Downregulation	SNAI1, NF*‐*κB1, E‐cadherin	Liu *et al*. (2012a)
182‐5p	Upregulated	FOXO3, MITF	Segura *et al*. (2009)
21‐5p	Upregulated	TIMP3	Martin del Campo *et al*. (2015), Yang *et al*. (2011)
Let‐7a‐5p	Downregulated	NRAS, integrin β3	Muller and Bosserhoff (2008)
34a‐5p	Downregulated	P53	Yamazaki *et al*. (2012)
	Downregulated	FLOT2	Liu *et al*. (2015b)
365a‐3p	Downregulated	NPL1	Bai *et al*. (2015)
7‐5p	Downregulated	IRS‐2/Akt	Giles *et al*. (2013)
125b‐5p	Downregulated	c‐Jun	Kappelmann *et al*. (2013)
	Downregulated	MLK3, MMK7	Zhang *et al*. (2014)
542‐3p	Downregulated	PIM1	Rang *et al*. (2016)
124‐3p	Downregulated	RLIP76	Zhang *et al*. (2016)
625‐5p	Downregulated	SOX2	Fang *et al*. (2017)
153‐3p	Downregulated	SNAI1	Zeng *et al*. (2017)
137	Downregulated	MITF, c‐Met, YB‐1, EZH2	Luo *et al*. (2013b)
339‐3p	Downregulated	MCL1	Weber *et al*. (2016)
214‐3p	Upregulated	ITGA5, ALCAM	Orso *et al*. (2016)
148b‐3p	Downregulated		
Immune response			
210‐3p	Downregulated	PTPN1, HOXA1, TP53I11	Noman *et al*. (2012)
30b‐5p/30d‐5p	Upregulated	GALNT7	Gaziel‐Sovran *et al*. (2011)
17‐5p	Downregulated	STAT3	Li *et al*. (2014a)
34a/c‐5p	Downregulated	ULBP2	Heinemann *et al*. (2012)
21‐5p, 29a‐3p, 142‐3p, 223‐3p	Upregulated	CSF1‐ETS2	Mathsyaraja *et al*. (2015)
34a/c‐5p, 499a/c	Downregulated	ULBP2	Heinemann *et al*. (2012)
28‐5p	Downregulated	TIM3, BTLA, PD‐1	Li *et al*. (2016)

^a^ MicroRNA names are updated to mirbase release 22 (March 2018).

As a technical note, we would like to emphasize that given the changes in mature miRNA naming that have occurred over the years (see miRBase database http://www.mirbase.org/help/nomenclature.shtml, Ambros *et al*., 2003), it was sometimes difficult to determine the identity of mature miRNAs in certain research articles, especially after the broader introduction of the −3p and −5p suffixes convention for mature miRNA naming as a substitute for the star (*) symbol for the less predominant form. Here, we decided to use theoriginal name of the mature miRNA, as used in the reference paper, but we provided an mirbase v.22 update name in the Tables. An mirbase tracker tool was used for miRNA name conversion (Van Peer *et al*., 2014).

#### 2.1.1 MicroRNAs involved in melanoma biology

Microphthalmia‐associated transcription factor (MITF) is the leading regulator of melanocyte development, survival and function (Levy *et al*., 2006). In melanoma, it was observed that MITF is both regulated by and regulates miRNAs.

The most cancer‐specific dysregulated miRNA in melanoma is miR‐211‐5p, which is indeed transcribed by MITF together with its host gene, melastatin (*TRPM1*), in human melanocytes. Several studies demonstrated that miR‐211 is one of the most differentially expressed miRNAs between melanoma cell lines and normal human melanocytes (Levy *et al*., 2010; Mazar *et al*., 2010; Xu *et al*., 2012). In primary melanoma, miR‐211 is downregulated, and it is downregulated even further in malignant melanoma. TRPM1/miR‐211 levels are frequently downregulated or lost during the transition from nevi to primary melanoma, and high TRPM1 levels correlate with longer disease‐free survival in primary melanoma patients (Hammock *et al*., 2006). MicroRNA is also involved in the regulation of cellular adhesion through the upregulation of NUAK1 (Bell *et al*., 2014). Overexpression of miR‐211 results in an increase in pigmentation via an increase in the total number of melanosomes and potentiates the pigmentation induced by vemurafenib by increasing the number of heavily pigmented stage IV melanosomes. The role of miR‐211 in melanotic melanoma cells is to contribute to a ‘normalization program’ activated by the inhibition of the ERK pathway: the resulting de‐repression of MITF promotes a switch from glycolysis to oxidative phosphorylation involving PGC1alpha and mitochondrial biogenesis. This induces a more differentiated phenotype mediated by TRPM1/miR‐211 and the melanin biosynthetic pathway (Vitiello *et al*., 2017).

It was observed that MITF is also regulated by miRNAs such as miR‐182 and miR‐137 which directly target MITF, leading to extracellular matrix degradation and, consequently, tumor cell migration and invasion (Bemis *et al*., 2008; Segura *et al*., 2009).

#### 2.1.2 MicroRNAs involved in cell proliferation

Melanoma cell proliferation can be influenced by miRNAs; in fact the dysregulation of some miRNAs can sustain and induce proliferative signals or repress growth‐suppressive pathways, thereby promoting melanoma carcinogenesis. Moreover, miRNAs can affect proliferation by regulating proteins involved in the cellular cycle.

One of the miRNAs involved in melanoma cell proliferation is miR‐21‐5p (formerly miR‐21). The miR‐21‐5p is upregulated in melanoma cell lines relative to melanocytes (Satzger *et al*., 2012), and in primary melanoma compared with benign nevi (Grignol *et al*., 2011). Upregulation of this miRNA is present in primary lesions with histological atypia and mitotic activity (Grignol *et al*., 2011). In addition, miR‐21‐5p is upregulated in metastatic melanoma compared with primary melanoma (Jiang *et al*., 2012). Increased expression of miR‐21‐5p affects proliferation, migration and apoptosis. Knockdown of miR‐21‐5p in melanoma cell lines reduces cell proliferation and migration, and promotes apoptosis by increasing the expression of programmed cell death 4 (PDCD4), phosphate and tensin homolog (PTEN) and BTG family member 2 (BTG2) (Yang *et al*., 2011).

Upregulation of miR‐155 occurs in primary melanoma compared with benign nevi (Grignol *et al*., 2011), in melanoma with positive sentinel lymph node biopsy compared with negative sentinel biopsy (Grignol *et al*., 2011), and in primary melanoma and metastatic melanoma compared with benign nevi (Philippidou *et al*., 2010; Segura *et al*., 2010). Contrary to what might be expected, *in vitro* experiments demonstrated that overexpression of miR‐155 results in inhibition of cellular proliferation and promotion of apoptosis (Levati *et al*., 2009) through targeting of v‐ski avian sarcoma viral oncogene homolog (SKI; Levati *et al*., 2011).

A study has found that miR‐135a, which promotes cell proliferation and the cell cycle, is upregulated in malignant melanoma tissue and cell lines. It was observed that ectopic expression of miR‐135a inhibits Forkhead box protein O1 (FOXO1) protein, leading to an upregulation of Cyclin D1 (CCND1) and downregulation of P21^Cip1^ and P27^Kip1^ through the AKT pathway (Ren *et al*., 2015).

Conversely, there are miRNAs that act as tumor suppressors and whose downregulation in cancer cells increases the proliferation rate: miR‐145, miR‐125b and miR‐206. Of these, miR‐145 is dysregulated in many solid tumors and is also downregulated in melanoma. In melanoma cell lines, it was observed that ectopic expression of miR‐145 inhibits cell growth by targeting c‐MYC (Noguchi *et al*., 2012a). In melanoma, miR‐125b is downregulated compared with normal skin (Holst *et al*., 2011). This downregulation was also demonstrated in melanoma cell lines relative to human epidermal melanocytes (Kappelmann *et al*., 2013; Zhang *et al*., 2014), in atypical nevi in comparison with common nevi (Holst *et al*., 2011) and in melanoma with lymph node involvement (N^+^) compared with melanoma without lymph node involvement (N0) (Glud *et al*., 2010). Experimentally, the overexpression of miR‐125b in melanoma cell line (Mel‐Juso) resulted in decreased proliferation and cell cycle arrest (Nyholm *et al*., 2014). A significant downregulation of miR‐206 was found in melanoma cells compared with normal melanocytes. This miRNA targets cyclin‐dependent kinase 4 (CDK4), cyclin D1 (CCND1) and cyclin C, and transfection of miR‐206 induces G1 arrest in multiple melanoma cell lines (Georgantas *et al*., 2014).

An important miRNA family that controls cell proliferation is the let‐7 family. The expression of let‐7a, let‐7b and let‐7d is significantly decreased in melanocytic nevi compared with primary melanoma (Schultz *et al*., 2008). The overexpression of miR‐let‐7b in melanoma cell lines decreased expression of cyclins D1, D3 and A, which are important for blocking the tumor‐suppressor retinoblastoma protein (Rb) and promoting proliferation in melanoma (Schultz *et al*., 2008).

The expression of miR‐193b was found to be 3.4‐fold lower in metastatic melanoma than in benign nevi. It was also observed that overexpression of miR‐193b in melanoma cell lines inhibits proliferation. In addition, it was demonstrated that miR‐193b directly targets CCND1 (Chen *et al*., 2010). It was suggested that dysregulation of this miRNA may contribute to melanoma progression. Similarly, miR‐137 is involved in the regulation of cell proliferation and is downregulated in melanoma cell lines from a stage IV patient compared with normal human melanocytes. It was demonstrated that miR‐137 inhibits proliferation mediated by tyrosine‐protein kinase Met (c‐Met), Y box binding protein 1 (YB1), MITF, and enhancer of zeste homolog 2 (EZH2) (Luo *et al*., 2013a). The expression of miR‐365 is lower in melanoma cells than in normal melanocytes, and it was observed that overexpression of miR‐365 inhibits proliferation and induces cell cycle arrest via inhibition of its targets, CCND1 and BCL2 (Zhu *et al*., 2018).

A study analyzed miR‐101 expression in melanoma cell lines from stage IV melanoma patients who had different survival times. The results suggest that survival might be favored by high levels of miR‐101. In fact, it was demonstrated that miR‐101 inhibits proliferation through the downregulation of MITF and EZH2 (Luo *et al*., 2013a). Despite this observation, miR‐101 is not considered to be a classical tumor suppressor because of its low expression in human melanocytes (NHEM).

In addition to miR‐211, there is a group of miRNAs, namely, miR‐203, ‐204 and ‐205, which show skin‐specific expression. All of these miRNAs have been found to be dysregulated in melanoma. Specifically, miR‐205 is downregulated in metastatic and primary melanomas compared with benign nevi (Xu *et al*., 2012). This finding was confirmed by Hanna *et al*. (2012), who also demonstrated that miR‐205 downregulation is associated with worse clinical outcome. Overexpression of miR‐205 in cell lines reduced anchorage‐independent colony formation, thereby reducing survival capability (Franken *et al*., 2006; Liu *et al*., 2012b), and is also correlated with zinc‐finger E‐box binding homeobox 2 (ZEB2) downregulation, E‐cadherin upregulation (Liu *et al*., 2012b) and suppression of cell proliferation via E2F1 and E2F5 targeting (Dar *et al*., 2011).

Both miR‐203 and 126/126* were found to be downregulated in melanoma, in particular in metastatic melanoma. Specifically, the downregulation of miR‐203 was inversely correlated with B lymphoma Mo‐MLV insertion region 1 homolog (BMI1) levels. It was demonstrated that miR‐203 inhibits the proliferation by targeting BMI1, thereby reducing invasion and tumor sphere formation (Chang *et al*., 2015). In addition, it was observed that ectopic expression of miR‐203 in melanoma cells reduced the expression of E2F3a and E2F3b, leading to the inhibition of cell growth and induction of cell cycle arrest and senescence (Noguchi *et al*., 2012b). It was experimentally demonstrated that restored expression of miR‐126/126* reduces cell proliferation, invasion *in vitro*, and melanoma growth and dissemination *in vivo*. The opposite effect was observed when miR‐126/126* were silenced, probably due to their direct action on two metalloproteases, namely ADAM9 and MMP7, which play a pivotal role in melanoma progression (Felli *et al*., 2013).

Another miRNA with a tumor suppressor role in melanoma is miR‐194. It was observed that the overexpression of miR‐194 inhibits cell proliferation through the PI3K/AKT/FoxO3a signaling pathway (Bai *et al*., 2017). In addition, another study observed that the inhibition of proliferation by miR‐194 can be due to the negative regulation of Rho guanine nucleotide exchange factor 2 (GEF‐H1) (Guo *et al*., 2016b). They also observed a negative association between miR‐194 expression and TNM stages.

There was significant downregulation of miR‐485‐5p in melanoma tissue and cell lines compared with corresponding controls. It was observed that the overexpression of miR‐485‐5p inhibits proliferation and invasion mediated by the downregulation of Frizzled7 (FZD7), indicating a role of miR‐485‐5p in the regulation of Wnt signaling (Wu *et al*., 2017).

Another miRNA linked to the Wnt pathway is miR‐136. The expression level of miR‐136 is decreased in mouse melanoma cells and is linked to the progression of melanoma. It was observed that miR‐136 acts as a tumor suppressor by inhibiting proliferation, migration and invasion, and promoting apoptosis. The action of miR‐136 is mediated by the inhibition of premelanosome protein (PMEL), resulting in the downregulation of the Wnt signaling pathway (Wang *et al*., 2017b).

Most of the functional studies on the role of miRNAs in melanoma were performed using melanoma cell lines *in vitro*. There are a few recent studies that used miRNA mimics or anti‐miRNA molecules to treat melanoma *in vivo*. All of them were based on the use of melanoma‐derived cells that were injected into immunocompromised mice. No *in vivo* miRNA study has yet been performed using genetically engineered mouse models of melanoma (Perez‐Guijarro *et al*., 2017) or patient‐derived xenografts (PDX) (Yan *et al*., 2018).

Zheng *et al*. (2018) found that overexpression of SOX10 promoted melanoma cell proliferation and chemotherapy resistance both *in vitro* and *in vivo*, and was associated with poor overall survival. They also demonstrated that miR‐31 could regulate tumor cell growth and chemosensitivity of melanoma cells by suppressing SOX10. The miR‐31–SOX10 axis mediates tumor growth and drug resistance through activation of the PI3K/AKT signaling pathway.

#### 2.1.3 MicroRNAs involved in apoptosis

Uncontrolled cell growth involves the loss of control of apoptosis. Certain proteins, including BCL2 and BCL2‐like 1, have anti‐apoptotic roles and can be the target of specific miRNAs. Furthermore, p53 activity is positively and negatively regulated by specific miRNAs (Feng *et al*., 2011; Liu *et al*., 2017). One of them is miR‐18b, which upregulates p53 by downregulating mouse double minute 2 homolog (MDM2); miR‐18b was found to be downregulated in melanoma compared with benign nevi and its downregulation was responsible for decreased p53 activity (Dar *et al*., 2013).

Overexpression of miR‐638 is reported in metastatic lesions compared with primary melanomas; it downregulates the TP53INP2 oncosuppressor and thereby protects melanoma cells from apoptosis and autophagy (Bhattacharya *et al*., 2015).

There are many miRNAs already known to be involved in the control of proliferation and cell cycle that also affect apoptosis. One of them is the oncomiR miR‐21 (Yang *et al*., 2011). Jiang *et al*. (2012) reported upregulation of PTEN upon miR‐21 inhibition, and also observed changes in B‐cell lymphoma 2 (BCL‐2) and phosphorylated RAC‐alpha serine/threonine‐protein kinase (pAKT). Furthermore, miR‐21‐5p expression can be induced by UV irradiation in human keratinocytes (Syed *et al*., 2013).

Compared with benign melanocytes, melanoma exhibits upregulated expression of miR‐4286, which promotes proliferation and protects from apoptosis. The use of miR‐4286 inhibitors leads to the alteration of miR‐4286 targets that are implicated in proliferation and apoptosis pathways: folylpolyglutamate synthase (FPGS), RNA polymerase I‐specific transcription initiation factor (RRN3), apelin (APLN), G‐protein‐coupled receptor 55 (GPR 55) and high‐mobility group A1 protein (HMGA1) (Komina *et al*., 2016).

A significant upregulation of miR‐15b is seen in melanoma compared with melanocytic nevi. High expression of miR‐15b is correlated with worse survival. Downregulation of miR‐15b inhibits cell proliferation and promotes apoptosis. It was observed that high levels of miR‐15b are associated with an increase of caspase 3 and 7, and annexin V, whereas Bcl‐2 was not induced. This suggests that miR‐15b may promote apoptosis independently of Bcl‐2 in melanoma cells (Satzger *et al*., 2010).

In addition to the earlier described role in cell proliferation, miR‐125b can also affect senescence and apoptosis. In fact, miR‐125b‐transfected cells showed increased levels of p27, p53 and p21, and consequently induced senescence (Nyholm *et al*., 2014).

The miRNA miR‐205 is considered a tumor suppressor for promoting apoptosis: Dar *et al*. (2011) suggested that the downregulation of miR‐205 in metastatic melanomas may lead to the activation of E2F transcription factor 1 (E2F1) and the inhibition of Rb.

Reuland *et al*. (2013) studied the role of miR‐26a in melanoma: they observed downregulation of this miRNA in melanoma cells compared with normal melanocytes. In addition, the replacement of miR‐26a promoted cell death by targeting directly the anti‐apoptotic protein silencer of death domains (SODD) (Reuland *et al*., 2013).

#### 2.1.4 MicroRNAs involved in invasion and metastasis

For the development of metastasis, it is necessary for the tumor to acquire the capacity to migrate and go through a de‐differentiation program called epithelial‐mesenchymal transition (EMT).

The upregulation of miR‐150, observed in primary and metastatic melanoma in comparison with congenital nevi (Segura *et al*., 2010), was implicated in cellular proliferation and cellular migration (Howard *et al*., 2013; Walker *et al*., 1998) through the activity of miR‐150 on targets such as v‐myb avian myeloblastosis viral oncogene homolog (MYB), early growth response 2 (EGR2) and neurogenic locus notch homolog protein 3 (NOTCH3), as well as on immune system‐related genes, cytokine signaling cascades and G‐proteins (Fleming *et al*., 2015; Howard *et al*., 2013; Kunz, 2013).

It has been reported that reducing miR‐211 expression using a miR‐211 specific ‘antagomir’ enhanced melanoma invasiveness 10‐fold. Conversely, overexpression of miR‐211 decreased the invasive potential of melanoma cells, but did not change the growth rate (Levy *et al*., 2010). The miR‐211 caused reduced expression of transforming growth factor beta (TGFβ), which furthered invasion and melanoma metastasis (Levy *et al*., 2010). Overexpression of miR‐211 can also result in decreased expression of brain‐specific homeobox/POU domain protein (BRN2) (Boyle *et al*., 2011) and ion channel KCNMA1 (Mazar *et al*., 2010), the upregulation of which is associated with increased cellular invasion in melanoma and other cancers.

In addition to its involvement in proliferation, miR‐101 inhibits the invasion of melanoma cells, likely due to the downregulation of its target MITF and EXH2 genes (Luo *et al*., 2013a).

The miR‐200 family plays an important role in cancer migration. The expression of the miR‐200 family is upregulated in melanoma and promotes tumor cell migration. In particular, the transfection of miR‐200c in melanoma cells induces an ameboid‐like invasion mode, with the cells assuming a round cell‐body phenotype. It was suggested that the effect could be due to the downregulation of MARCKS, which is important for the formation of cell protrusions. On the other hand, miR‐200a promotes the protrusion‐associated elongated invasion mode because it reduces actomyosin contractility, which is a feature of a rounded phenotype (Elson‐Schwab *et al*., 2010).

In melanoma, miR‐203 is downregulated and consequently its target BMI1 is upregulated. It was observed that overexpression of miR‐203, in addition to suppressing proliferation, leads to the inhibition of the invasiveness in melanoma (Chang *et al*., 2015).

Low expression levels of miR‐9 were seen in metastatic melanoma compared with primary melanoma. In melanoma, miR‐9 acts as a tumor suppressor. Its role consists in metastasis inhibition by the downregulation of Zinc‐finger protein SNAI1 (Snail1) and nuclear factor kappa‐light‐chain‐enhancer of activated B cells (NF‐κB1) and by upregulation of E‐cadherin (Liu *et al*., 2012a).

In melanoma cell lines, miR‐182 was found upregulated and its overexpression promoted metastasis by repressing FOXO3 and MITF (Segura *et al*., 2009).

It was demonstrated that miR‐21 upregulation promotes invasiveness through the downregulation of the tissue inhibitor of metalloproteinase‐3 (TIMP3) (Martin del Campo *et al*., 2015; Yang *et al*., 2011). In this study, mice treated with anti‐miR‐21 molecules showed a significant reduction of TIMP3 expression and tumor growth and invasiveness.

It has been reported that let‐7a is downregulated in melanoma cells compared with melanocytes (Muller and Bosserhoff, 2008). Moreover, this miRNA targets neuroblastoma RAS viral oncogenes homolog (NRAS) and human integrin β3, which have a well‐documented role in melanoma progression and invasion. The amount of let‐7a has been linked to upregulation of integrin β3 in melanoma cells (as shown by transient *in vitro* overexpression and luciferase assays), indicating that this miRNA has a tumor‐suppressive role in melanoma (Muller and Bosserhoff, 2008).

The miR‐34a is part of the miR‐34 family (miR‐34a/b/c) that is regulated by p53. In human melanoma cell lines, the overexpression of the miR‐34 family inhibits the growth and invasion of cells expressing wild‐type p53 gene (Yamazaki *et al*., 2012). There is significant downregulation of miR‐34a in metastatic melanoma compared with *in situ* melanoma, nevi and normal melanocytes. The high expression of miR‐34a also inhibits proliferation and metastasis by targeting flotillin 2 (FLOT2) (Liu *et al*., 2015b).

A strong downregulation of miR‐365 was shown in malignant melanoma tissue and cells lines. The ectopic expression of miR‐365 inhibits growth, invasion and metastasis in malignant melanoma by directly targeting neuropilin‐1 (NPL1) (Bai *et al*., 2015).

Giles *et al*. (2013) studied the role of miR‐7‐5p in melanoma. They observed a downregulation of miR‐7‐5p in metastatic melanoma‐derived cell lines compared with primary melanoma cells. Furthermore, the ectopic expression of miR‐7‐5p suppresses cell migration and invasion by directly targeting insulin receptor substrate‐2 (IRS‐2), and the inhibition of IRS‐2 reduced the **activity of** protein kinase B (AKT) (Giles *et al*., 2013).

MicroRNA miR‐125b is downregulated in melanoma especially in metastatic melanoma; miR‐125b acts as tumor suppressor and decreases cell migration (Kappelmann *et al*., 2013). This could be mediated by the downregulation of the transcription factor c‐Jun (Kappelmann *et al*., 2013) and serine/threonine kinase mixed lineage kinase (MLK3) protein and mitogen‐activated protein kinase kinase 7 (MMK7), which are direct targets of miR‐125b (Zhang *et al*., 2014).

Melanoma cell lines and tissues showed a downregulation of miR‐542‐3p. It was observed that the ectopic expression of miR‐542‐3p suppresses tumor cell migration, invasion and EMT through the inhibition of its target, the proto‐oncogene serine/threonine protein kinase (PIM1) (Rang *et al*., 2016). The expression levels of miR‐124 were negatively correlated with the advanced stage of melanoma. The tumor suppressor effect of miRNA‐124 consisted in the suppression of proliferation and invasion mediated by the inhibition of its target RLIP76, which is overexpressed in melanoma cell lines (Zhang *et al*., 2016). MicroR‐625 was found downregulated in malignant melanoma and it was shown that its ectopic expression inhibits proliferation and migration in malignant melanoma. In particular, in malignant melanoma, the expression level of miR‐625 was inversely correlated with that of transcription factor SOX‐2 (SOX2), suggesting that the anti‐tumor action of miR‐625 is at least partially mediated by the inhibition of SOX2 (Fang *et al*., 2017).

Also, miR‐153‐3p is downregulated in melanoma tissue and cell lines. In particular, it was observed that miR‐153‐3p modulates cell proliferation and invasion by the inhibition of the expression of SNAI1, which is a zinc‐finger transcription factor involved in the promotion of the EMT (Zeng *et al*., 2017).

MicroRNA miR‐137 acts as tumor suppressor; in fact, the expression of miR‐137 in melanoma leads to the inhibition of the proliferation and invasion by the downregulation of its targets, including MITF, c‐Met, Y‐box‐binding protein 1 (YB‐1) and enhancer of zeste homolog 2 (EZH2). In addition, a correlation was observed between miR‐137 expression and prognosis; low levels of miR‐137 are associated with a short survival in stage IV melanoma patients (Luo *et al*., 2013b).

Weber *et al*. (2016) performed a very interesting study where they tested a large panel of miRNA mimics to assess their effect on melanoma A375 cell line invasion capability. They identified the miRNAs that were most effective in promoting cell invasion (miR‐576‐5p, miR‐21, miR‐214 and miR‐182) and those that were most effective in preventing cell invasion (miR‐339‐3p, miR‐211, miR‐101, miR‐126‐3p and ‐5p). They then tested the effect of miR‐339‐3p *in vivo* by performing lung colonization assays in immunodeficient NSG mice. They found that mice injected with A375 cells overexpressing miR‐339‐3p carried significantly fewer tumor nodules compared with control mice, consistent with the inhibitory effect of miR‐339‐3p on tumor cell invasion *in vitro*. In addition, they blocked miR‐339‐3p using an antagomir and found an increase of melanoma cell invasion, an effect that could be phenocopied by RNAi‐mediated silencing of MCL1, which is a target of miR‐339‐3p.

Orso *et al*. (2016) studied the role of miR‐214 and miR‐148b in the process of metastatic dissemination. Depleting miR‐214 or elevating miR‐148b blocked the dissemination of melanoma, an effect that could be accentuated by their dual alteration (Orso *et al*., 2016). In fact, they demonstrated that the dual alteration suppresses the passage of malignant cells through the blood vessel endothelium by reducing the expression of the cell adhesion molecules ITGA5 and ALCAM; furthermore, single or combined miR‐214 downregulation and miR‐148b upregulation in tumor cells inhibits metastasis formation in mice.

#### 2.1.5 MicroRNA involved in immune response

One hallmark of melanoma biology is immune evasion. This can be induced by senescence, alterations in antigen presentation, interference with regulatory T cells or hypoxia (Noman *et al*., 2012). The role of the immune system in melanoma is widely known and immunotherapies based on immune checkpoint (CTLA4 and PD1) inhibitors have been developed, such as anti‐CTLA4 and anti‐PD1 (Postow *et al*., 2015). Recently, some evidence of the immune suppressive/evasive effects of families of miRNAs.

Immune escape can be promoted by a hypoxic microenvironment (Noman *et al*., 2011). In melanoma, hypoxia‐induced miR‐210 expression level resulted in the escape from cell lysis by antigen‐specific cytotoxic T lymphocytes (CTL or CD8^+^ T cells) (Noman *et al*., 2012). In the same study, it was observed that in hypoxic cells, miR‐210 targets and inhibits protein tyrosine phosphatase non‐receptor type 1 (PTPN1), homeobox protein Hox‐A1 (HOXA1) and tumor protein p53‐inducible protein 11 (TP53I11; Noman *et al*., 2012).

MicroRNA‐30b and ‐30d act as immunosuppressive miRNAs. It was observed that miR‐30b and miR‐30d are upregulated in melanoma, and their increased expression level correlates with an advanced stage, metastatic potential and a worse prognosis. The ectopic expression of miR‐30b/30d leads to the downregulation of GalNAc transferase GALNT7, resulting in suppression of immune cell activation and recruitment mediated by a high level of immunosuppressive cytokine IL‐10 (Gaziel‐Sovran *et al*., 2011).

Other miRNAs that are able to influence the immune response are miR‐34a/c. In particular, miR‐34a/c control the expression of UL16 binding protein 2 (ULBP2), which is a ligand for NK cell immunoreceptor NKG2D. The downregulation of miR‐34a/c, which occurs frequently in cancer, leads to an upregulation of ULBP2, thus paradoxically resulting in an increased tumor‐immune surveillance by natural killer (NK) cells (Heinemann *et al*., 2012).

The microRNAs miR‐21, miR‐29a, miR‐142‐3p and miR‐223 are induced in macrophages by activation of CSF1‐ETS2 pathway and can influence melanoma growth and metastasis. Consistently, miR‐21 and miR‐29a are upregulated in specific suppressive myeloid populations in mouse bone marrow and patient blood during melanoma metastatic progression (Mathsyaraja *et al*., 2015). MicroRNAs miR‐34a/c and miR‐499a/c bind to the 3′‐UTR of ULBP2, a ligand of NKG2D receptor that activates NK cells against the tumor (Heinemann *et al*., 2012).

Ultraviolet radiation is the main risk factor for CM. Experimental models for UV‐induced melanoma have highlighted that UVR‐induced inflammation can promote immune evasion (Hodis *et al*., 2012). Recently, the effect of UV exposure on miRNA expression in melanocytic nevi was explored (Bell *et al*., 2018) and a depth‐related signature was identified. Another recent study demonstrated that 14 miRNAs are altered after UV exposure, leading to the increase of immune evasive molecules such as CCL2, CCL8, PD1 and B7H2 (Sha *et al*., 2016).

MicroRNAs can also be involved in immune checkpoint regulation; in fact, exhausted T cells in melanoma show a downregulation of miR‐28 expression. MicroRNA miR‐28 binds the 3′‐UTR of TIM3, BTLA and PD‐1. When miR‐28 mimics were administered to exhausted T cells, the phenotype reverted and IL‐2 and tumor necrosis factor (TNF)‐α production restored (Li *et al*., 2016). Because of PD‐L1 regulation, miR‐17‐5p has also been proposed as a prognostic biomarker. BRAF‐ and MEK‐inhibitor‐resistant melanoma cell lines showed increased expression of PD‐L1, which is inversely correlated with miR‐17‐5p expression. The authors demonstrated that PD‐L1 is a direct post‐transcriptional target of miR‐17‐5p (Audrito *et al*., 2017).

### 2.2 MicroRNAs as diagnostic or prognostic biomarkers

#### 2.2.1 Circulating miRNAs in melanoma

An important characteristic of miRNAs is that they are released by the tumor in the bloodstream. Tumor‐derived endogenous miRNAs are very stable in the blood and are resistant to RNAse activity. It is possible to analysis miRNA levels in blood samples in a non‐invasive way, performing a ‘liquid biopsy’. In 2008, it was reported for the first time that circulating miRNAs could be used as biomarkers in patients with solid tumors (Mitchell *et al*., 2008). Circulating miRNAs can be used for cancer diagnosis and prognosis (Ferracin and Negrini, 2015). The circulating miRNA profile is different from that of tumors and relies strongly on the protocol used for sample processing, probably because of the different amount of extracellular vesicles (EVs) retained during plasma/serum preparation. Indeed, exosomes and EVs in general are one of the main repositories of miRNAs in the blood (Cheng *et al*., 2014).

Some pilot studies for the analysis of the entire spectrum of circulating miRNAs by microarray and small RNA sequencing (Ferracin *et al*., 2015) or quantitative PCR (Greenberg *et al*., 2013; Stark *et al*., 2015b) have been performed in the past few years. Ferracin *et al*. (2015) demonstrated the potential of miR‐320a as a plasma melanoma biomarker in comparison with other main types of solid tumors and healthy subjects. In contrast, in their study, Greenberg *et al*. (2013) showed that there was a significant reduction of circulating miR‐29c and miR‐324‐3p in the serum of melanoma patients compared with healthy subjects. The decrease of miR‐29c expression was also observed in stage III/IV compared with stage I/II melanoma tumors and it was associated with a poor prognosis in CM (Nguyen *et al*., 2011). Stark *et al*. (2015b) performed a screening on the level of 233 miRNAs in melanoma and healthy controls and across melanoma stages. They identified a panel of 17 miRNAs (MEL‐miR‐17 signature) that was correlated with stage, recurrence and survival and with a predictive potential. In that study, they further selected a panel of seven miRNAs that was able to detect the presence of melanoma with high sensitivity (93%) and specificity (82%). In this panel, miR‐4487, miR‐4706, miR‐4731, miR‐509‐3p and miR‐509‐5p were reduced, whereas miR‐16 and miR‐211 (stage IV only) were increased in melanoma.

Another panel was proposed by Margue *et al*. (2015), who found that in serum samples of melanoma patients the levels of miR‐122‐5p and miR‐3201 were higher than in serum samples of healthy people.

In another study, the quantification of 21 miRNAs in the plasma of melanoma patients and healthy subjects identified five miRNAs that can be used as diagnostic markers. In particular, the upregulation of circulating miR‐149‐3p, miR‐150‐5p and miR‐15b‐5p, and the downregulation of circulating miR‐193a‐3p and miR‐524‐5p were associated to melanoma (Fogli *et al*., 2017).

Armand‐Labit *et al*. (2016) demonstrated that the detection of miR‐185 and miR‐1246 in plasma discriminated patients with metastatic melanoma from healthy individuals with a sensitivity of 90.5% and specificity of 89.1%.

Circulating miRNA can also be used to provide prognostic information. Friedman *et al*. (2012) proposed a panel of five circulating miRNAs to identify melanoma patients with high risk of recurrence. In particular, they found that in melanoma patients’ serum samples, low levels of miR‐15b and miR‐33a, and elevated levels of miR‐150, miR‐199a‐5p and miR‐424 were associated with a high risk of recurrence.

A predictive panel formed by miR‐150‐5p, miR‐30d‐5p, miR‐15b‐5p and miR‐425‐5p in conjunction with the pathologic stage was superior for predicting recurrence‐free and overall survival when compared with conventional staging criteria. In addition, it was shown that miR‐15b levels can be useful for early monitoring for recurrence in melanoma patients (Fleming *et al*., 2015).

Shiiyama *et al*. (2013) proposed a panel of six miRNAs to identify metastatic melanoma. In fact, the expression of miR‐150‐5p, miR‐9‐5p, miR‐145‐5p, miR‐155‐5p, miR‐203 and miR‐205‐5p was significantly different in metastatic and non‐metastatic melanoma patients.

In a case–control study, it was found that the expression of circulating miR‐16 can be used as a prognostic biomarker. Expression levels of miR‐16 were lower in serum of melanoma patients than in serum of cancer‐free controls. Furthermore, it was shown that the decrease of miR‐16 was correlated with an increase in tumor thickness, ulceration status, AJCC stage, and tissue Ki‐67 expression (Guo *et al*., 2016a).

- A comparison of miR‐206 levels between 60 patients with melanoma and 30 healthy controls showed that serum levels of miR‐206 are significantly lower in melanoma. In addition, a correlation between miR‐206 levels and prognosis was reported. In fact, it was observed that patients with low serum miR‐206 levels present two or more sites of metastases and had a shorter 5‐year overall and disease‐free survival than melanoma patients with a high miR‐206 level (Tian *et al*., 2015).
- Plasma levels of miR‐21 were found to be elevated in melanoma in two independent studies (Ferracin *et al*., 2015; Saldanha *et al*., 2013). Plasma levels of miR‐210 were higher in individuals with melanoma and an increased level of miR‐210 predicted disease recurrence. Furthermore, miR‐210 increase in plasma was correlated with a poor prognosis (Ono *et al*., 2015).

MicroRNA miR‐221‐5p was shown to be increased in the serum of patients with metastatic melanoma, decreasing after excision and increasing with disease recurrence (Kanemaru *et al*., 2011).

The level of miR‐125b‐5p in serum‐derived exosomes of 21 patients with advanced melanoma was compared with that in 16 disease‐free patients and 19 healthy volunteers; miR‐125b‐5p expression was reduced in exosomes of individuals with an active disease (Alegre *et al*., 2014).

Xiao *et al*. (2016) demonstrated that melanoma cell‐derived exosomes actively interact with normal melanocytes. They also suggest that melanoma cell‐derived exosomes may promote the EMT‐resembling process through autocrine/paracrine signaling, creating a tumor‐supportive microenvironment, through the action of miR‐191 and let‐7a.

Pfeffer *et al*. (2015) investigated miRNA signatures of plasma‐derived exosomes from familial and sporadic melanoma patients and unaffected family members. They demonstrated that miR‐17, miR‐19a, miR‐21, miR‐126 and miR‐149 are expressed at higher levels in plasma‐derived exosomes from patients with metastatic melanoma. They then studied plasma from genetically predisposed familial melanoma patients with/without evidence of disease. They did not found differences between CDKN2A mutation carriers and controls.

Lunavat *et al*. found that inhibition of BRAFV600E with vemurafenib and dabrafenib was associated with increased secretion of EVs from melanoma cells. They observed the specific increase of miR‐211‐5p after treatment in cells and EVs both *in vitro* and *in vivo*. This result indicated that target therapies change the RNA cargo in tumor‐derived EVs. In addition, miR‐211‐5p forced expression reduced sensitivity to BRAF inhibitors and decreased its efficiency in melanoma cell lines (Lunavat *et al*., 2017).

MicroRNA miR‐222 is involved in melanoma development by controlling tumor progression through the down‐modulation of its target genes: p27Kip1/CDKN1B, c‐KIT and c‐FOS (Felicetti *et al*., 2008). It was later discovered that miR‐222 can be detected in exosomes from human melanoma cell lines established from primary or metastatic tumors. It has been also demonstrated that miR‐222 can be transferred between cells, resulting in the capability of promoting malignancy through p27Kip1 inhibition and consequent PI3K/AKT pathway activation in the recipient cells (Felicetti *et al*., 2016).

A summary of the miRNAs that can be detected in the circulation in melanoma is presented in Table 2.

**Table 2:** Circulating miRNAs with a diagnostic, prognostic or functional impact in melanoma

miRNA	Significance	Levels in melanoma patients vs. healthy subjects	References
320a	Diagnostic marker	Increased	Ferracin *et al*. (2015)
29c, 324‐3p	Diagnostic marker	Decreased	Greenberg *et al*. (2013)
4487, 4706, 4731, 509‐3p, 509‐5p	Diagnostic marker	Decreased	Stark *et al*. (2015b)
16‐5p, 211‐5p		Increased (only stage IV)	
29c‐3p	Staging (III/IV vs. I/II)	Decreased	Nguyen *et al*. (2011)
122‐5p, 3201	Diagnostic marker	Increased	Margue *et al*. (2015)
149‐3p, 150‐5p, 15b‐5p	Diagnostic marker	Increased	Fogli *et al*. (2017)
193a‐3p, 524‐5p		Decreased	
185‐5p, 1246	Diagnostic marker	Increased	Armand‐Labit *et al*. (2016)
15b‐5p, 33a‐5p	Prognostic marker	Decreased	Friedman *et al*. (2012)
150‐5p, 199a‐5p, 424‐5p		Increased	
150‐5p, 30d‐5p, 15b‐5p, 425‐5p	Prognostic marker		Fleming *et al*. (2015)
150‐5p, 9‐5p, 145‐5p, 155‐5p, 203, 205‐5p	Metastasis marker		Shiiyama *et al*. (2013)
16‐5p	Prognostic marker	Decreased	Guo *et al*. (2016a)
206	Diagnostic and prognostic marker	Decreased	Tian *et al*. (2015)
21‐5p	Diagnostic and prognostic marker	Increased	Ferracin *et al*. (2015), Saldanha *et al*. (2013)
210	Diagnostic and prognostic marker	Increased	Ono *et al*. (2015)
221‐5p	Prognostic marker	Increased	Igoucheva and Alexeev (2009), Kanemaru *et al*. (2011)
125b‐5p	Diagnostic marker	Decreased	Alegre *et al*. (2014)
191 and let‐7a	EMT	Released in exosomes	Xiao *et al*. (2016)
17‐5p, 19a‐3p, 21‐5p, 126‐3p, 149‐5p	Diagnostic marker	Released in exosomes	Pfeffer *et al*. (2015)
211–5p	Therapy resistance	Released in exosomes	Lunavat *et al*. (2017)
222‐3p	Malignant transformation	Released in exosomes	Felicetti *et al*. (2016)

#### 2.2.2 MicroRNAs in prognosis prediction and drug resistance

The dysregulated expression of specific miRNAs in melanoma cells could serve as a prognostic biomarker for the patients or interfere with their response to treatments (Table 3). Expression of miR‐21‐5p is an important prognostic factor in melanoma, where its increased expression was correlated with higher tumor stage and worst survival (Jiang *et al*., 2012). Galasso *et al*. (2018) demonstrated that the loss of miR‐204 is common in melanomas with NRAS sole mutation but is less frequent in those harboring CDKN2A mutations. Additionally, miR‐204 was associated with a better prognosis in two independent melanoma cohorts and its exogenous expression led to growth impairment in melanoma cell lines (Galasso *et al*., 2018).

**Table 3:** MicroRNAs (miRNAs) involved in prognosis prediction and drug resistance

miRNA	Significance	Expression	Reference
21‐5p	Prognostic marker	Upregulated in the worse prognosis group	Jiang *et al*. (2012)
204‐5p	Prognostic marker	Downregulated in NRASmut	Galasso *et al*. (2018)
150‐5p, 455‐3p, 145‐5p, 342‐3p, 497‐5p, 155, 342‐5p, 143‐3p, 193a‐3p, 146b‐5p, 28‐3p, 10b‐5p, 193b‐3p, 28‐5p, 142‐5p, 143‐5p, 126‐3p, 214‐3p	Prognostic marker	Upregulated in the better prognosis group	Segura *et al*. (2010)
34a‐5p, 100‐5p, 125b‐5p	Resistance to BRAF inhibitors	Upregulated in resistant cells	Vergani *et al*. (2016)
514a‐3p	Resistance to BRAF inhibitors	Upregulated in resistant cells	Stark *et al*. (2015a)
200c‐3p	Sensitivity to BRAF inhibitors	Upregulated in resistant cells	Liu *et al*. (2015a)
579‐3p	Resistance to BRAF/MEK inhibitors	Downregulated in resistant cells	Fattore *et al*. (2016)

Segura *et al*. established a signature of 18 miRNAs associated with metastatic melanoma survival. In particular, they showed that metastatic patients had a longer survival when the metastasis was overexpressing these miRNAs (miR‐150, mir‐455‐3p, miR‐145, miR‐342‐3p, miR‐497, miR‐155, miR‐342‐5p, miR‐143, miR‐193a‐3p, miR‐146b‐5p, miR‐28‐3p, miR‐10b, miR‐193b, miR‐28‐5p, miR‐142‐5p, miR‐143*, miR‐126 and miR‐214) (Segura *et al*., 2010). In addition, they proposed a reduced 6‐miRNA panel (miR‐150, miR‐455‐3p, miR‐145, miR‐497, miR‐155, miR‐342‐3p) to stratify stage III patients according to prognosis and also demonstrated its validity in primary tumors.

Several miRNAs can affect drug resistance in melanoma. It was observed that melanoma could acquire resistance against BRAF inhibitors by altering the pattern of cytokine production. The treatment with BRAF inhibitor vemurafenib led to an increase of CCL2, which acts as an autocrine growth factor in melanoma. CCL2 in turn upregulates miR‐34a, miR‐100 and miR‐125b (Vergani *et al*., 2016). In that study, high levels of these miRNAs were associated with apoptosis inhibition and drug resistance. The simultaneous inhibition of all the three miRNAs restored sensitivity to BRAF inhibitors by increasing apoptosis.

Another study observed that in melanoma, miR‐514 contributed to the sensitivity of BRAF inhibitors. Specifically, miR‐514 targets NF1 and leads to the maintenance of MAPK pathway activation. In this way, the overexpression of miR‐514 contributes to the resistance to BRAF inhibitors (Stark *et al*., 2015a).

Liu *et al*. (2015a) investigated the role of miR‐200c in resistance of BRAF inhibitors. They observed a downregulation of miR‐200c in resistant melanoma tumors and cell lines, with a consequent upregulation of its targets, including BMI1, ZEB2, TUBB3, ABCG5 and MDR1. The study showed that the overexpression of miR‐200c restores the sensitivity of BRAF inhibitors through the inhibition of PI3K/AKT and MAPK signaling pathways.

Fattore *et al*. (2016) found that the expression of miR‐579‐3p is a prognostic factor; in particular, low levels of miR‐579‐3p are associated with a poor prognosis. They observed that the expression levels decrease from nevi to stage III/IV melanoma samples and also that melanoma cell lines resistant to BRAF/MEK inhibitors showed a downregulation of this miRNA. The overexpression of miR‐579‐3p altered the drug sensitivity in melanoma cell lines.

### 2.3 MicroRNA editing in melanoma

RNA editing is the post‐translational process that changes the sequence of a transcribed RNA. This event is mediated by specific enzymes, including members of double‐stranded RNA‐specific adenosine deaminase (ADAR) and activation‐induced cytidine deaminase or its relative APOBEC cytidine deaminase families, which can be dysregulated in cancer (Porcellini *et al*., 2018). Specifically, mature miRNA nucleotides can be subjected to hydrolytic deamination of adenosine to inosine (A‐to‐I) or C‐to‐U conversion. In melanoma, a downregulation of ADAR1 in metastatic tumors was reported and correlated with reduced A‐to‐I editing of miR‐455‐5p, miR‐324‐5p and miR‐378a‐3p (Shoshan *et al*., 2015). The changes in miR‐455‐5p editing sites modified its activity on the target genes and conferred completely different biological functions on this miRNA.

Velazquez‐Torres *et al*. (2018) studied the effect of ADAR1 hypo‐editing in metastatic melanoma cells. Micro (mi)R‐378a‐3p undergoes A‐to‐I modification only in the non‐metastatic melanoma cells. The target of miR‐378a‐3p is the oncogene PARVA, but the gene is preferentially downregulated by the edited form of miR‐378a‐3p. In melanoma cells, the expression of α‐parvin and ADAR1 is inversely correlated. When they transfected the WT and edited form of miR‐378a‐3p in SB2 cells, α‐parvin expression reduction was observed only upon edited miR‐378a‐3p transfection.

Nemlich *et al*. (2013) studied metastatic melanoma cells that exhibit significant downregulation of ADAR1‐P110 and ADAR1‐P150 as compared with normal melanocytes, nevi and primary melanoma tumors. They reported that miR‐17‐5p and miR‐432 are direct, independent, endogenous cellular regulators of ADAR1. They also observed that the upregulation of miR‐21‐5p (by silencing) and the downregulation of miR‐34a (by forced expression) seems partially to reverse the enhanced proliferation of ADAR1‐KD cells. They also showed that amplification of the genomic segment encoding miR‐17‐5p occurs frequently in melanoma, facilitating the malignant phenotype by directly targeting ADAR1. In addition, the overexpression of miR‐432 in melanoma can be attributed to frequent genomic amplification and aberrant hypomethylation patterns of the DLK1‐DIO3 locus on chromosome 14.

### 2.4 Long non‐coding RNA dysregulation in melanoma

Besides small ncRNAs, several recent studies described the dysregulation and cancer‐promoting role of specific lncRNAs in CM (Table 4). From these studies, some lncRNAs were associated with stage (Yang *et al*., 2018) or metastasis (Wang *et al*., 2017a). Recently, two prognostic lncRNA signatures were proposed (Chen *et al*., 2017b; Yang *et al*., 2018), demonstrating the potential of lncRNA in melanoma classification. Functionally, the mechanisms used by two melanoma‐specific lncRNAs (SAMMSON and TYRP1) to promote tumor growth were recently described (Gilot *et al*., 2017; Leucci *et al*., 2016). The lncRNA SAMMSON is located in a genomic region that is amplified in melanoma and interacts with the mitochondrial protein p32 in regulating the survival of melanoma cells (Leucci *et al*., 2016). TYRP1 mRNA, independently of its protein‐coding activity, sequestering through its 3′‐UTR a microRNA (miR‐16) and thus dampening the tumor suppressor activity of miR‐16 itself (Gilot *et al*., 2017).

**Table 4:** Long non‐coding RNAs (lncRNAs) dysregulated in human cutaneous melanoma

lncRNA	Functional role	Expression in melanoma	References
SAMMSON	Interacts with p32	Upregulated	Leucci *et al*. (2016)
TYRP1	Sponge for miR‐16	Upregulated	Gilot *et al*. (2017)
SPRY4‐IT1	Melanoma cell growth and invasion	Upregulated	Khaitan *et al*. (2011), Mazar *et al*. (2014)
SPRY4‐IT1	Diagnostic and prognostic marker in serum	Upregulated	Liu *et al*. (2016)
LLME23	PSF binding	Upregulated	Wu *et al*. (2013)
UCA1	Prognostic marker	Upregulated	Tian *et al*. (2014), Wei *et al*. (2016)
	Target of miR‐507	Upregulated	Wei *et al*. (2016)
MALAT‐1	Target miR‐183 and ITGB1	Upregulated	Sun *et al*. (2017)
	Prognostic marker	Upregulated	Tian *et al*. (2014)
39 lncRNAs panel	Target BRAF^V600E^		Flockhart *et al*. (2012)
BANCR	Cell Migration	Upregulated	Li *et al*. (2014b)
ANRIL	CDKN2A/B germlines deletion	Upregulated	Sarkar *et al*. (2017)
PVT1	Cell proliferation and metastasization	Upregulated	Chen *et al*. (2017a, 2018)

Sprouty4‐intronic transcript 1 (SPRY4‐IT1) is one of the first described lncRNAs associated with melanoma; it has been reported to promote melanoma cell growth and invasion and inhibit apoptosis by altering lipid metabolism (Khaitan *et al*., 2011; Mazar *et al*., 2014). Normally, this lncRNA is expressed at low levels in human melanocytes but it is highly upregulated in human melanoma cells (Khaitan *et al*., 2011). Expression levels of this lncRNA have been evaluated in plasma of melanoma patients and matched controls, showing that patients have higher levels of SPRY4‐IT1 compared with healthy controls, associated with tumor site, tumor stage and poor prognosis (Liu *et al*., 2016).

Other lncRNAs involved in melanoma cell proliferation are LLME23, UCA1 and MALAT1 (Wei *et al*., 2016; Wu *et al*., 2013). Upregulation of LLME23 was detected in human melanoma cell lines and it was found to bind the protein‐associated splicing factor PSF, a well‐known tumor suppressor; by binding to PSF, LLME23 was able to promote the expression of the proto‐oncogene RAB23, a RAS‐related small GTPase (Wu *et al*., 2013). Moreover, LLME23 silencing reduced tumor growth *in vivo*.

UCA1 is upregulated in human melanoma tissues and cell lines and is involved in tumor cell proliferation, migration and invasion. Moreover, this lncRNA significantly increases with stages (Tian *et al*., 2014). UCA1 has a binding site for miR‐507, suggesting a co‐regulation of these two ncRNAs. A study on primary melanoma, metastatic melanoma and nevi from patients and melanoma cell lines showed that the UCA1 level is increased in primary and metastatic melanoma, as well as in cell lines, compared with nevi (Wei *et al*., 2016). The same authors also demonstrated a negative correlation between UCA1 and miR‐507, and that UCA1 silencing decreases the levels of FOXM1 by releasing miR‐507.

MALAT‐1 was demonstrated to increase progressively in melanoma progression in a cohort of 63 primary melanomas, adjacent normal tissue and metastatic lesions (Tian *et al*., 2014). MALAT‐1 promotes cell proliferation and invasion through a complex interaction with miR‐183 and integrin β1 (ITGB1) (Sun *et al*., 2017).

In an RNA sequencing study by Flockhart *et al*. (2012) the authors described a panel of 39 lncRNAs regulated by BRAFV600E in melanoma; the most significant was BRAF‐activated non‐coding RNA (BANCR). BANCR regulates a set of genes involved in cell migration, including the chemokine CXCL11, and can promote melanoma proliferation via activation of ERK1/2 and JNK MAPK pathway both *in vitro* and *in vivo* (Li *et al*., 2014b).

ANRIL (antisense non‐coding RNA in the INK4A locus) is a lncRNA first identified in familiar melanoma with CDKN2A/B (INK4B‐ARF‐INK4A) germline mutations (Sarkar *et al*., 2017). ANRIL is located in chromosome 9p21, nearby *CDKN2A/B* genes, and SNPs in this region have been associated with human diseases, e.g. coronary disease, stroke, diabetes, melanoma and glioma (Congrains *et al*., 2013). ANRIL presents different linear and circular isoforms due to alternative splicing, with different functional roles in melanoma (Sarkar *et al*., 2017). The main function of this lncRNA is to mediate the repression of the CDKN2A/B locus by association with polycomb repressor complexes (PRC1 and PRC2) involved in the methylation‐mediated control of histone3 (Richtig *et al*., 2017; Yap *et al*., 2010). ANRIL silencing was able to restore the proper expression of CDKN2A and B in a melanoma xenograft model (Xu *et al*., 2016).

The role of plasmacytoma variant translocation 1 (PVT1) as a regulator of cell proliferation and metastasis has been studied in melanoma (Chen *et al*., 2017a, 2018). PVT1 is overexpressed in melanoma samples and correlates with tumor stage. This association was also confirmed in plasma samples, underlining the possibility of using this lncRNA as a detection biomarker (Chen *et al*., 2017a). In addition, the authors suggest a role of PVT1 in the regulation of miR‐200c expression.

Long ncRNAs could be used as targets for melanoma treatment. Leucci *et al*. (2016) demonstrated that the intravenous administration of SAMMSON‐specific antisense oligonucleotide *in vivo* in combination with BRAF inhibitor dabrafenib in a melanoma patient‐derived xenograft (PDX) significantly induced apoptosis, reducing the tumor growth, whereas the administration of dabrafenib alone only inhibited tumor growth.

## 3 Concluding remarks and future perspectives

Cutaneous melanoma typically arises on sun‐exposed skin because of the progressive accumulation of UV radiation‐induced genetic alterations. These chronically sun‐damaged (CSD) melanomas are very different from non‐CSD melanomas (Shain and Bastian, 2016). UV exposure induces specific genetic alterations in melanocytes (e.g. prevalence of C‐to‐T transition) and a generally high tumor mutational burden, both in coding and non‐coding regions of the genome (Hayward *et al*., 2017). This in turn generates a broad range of genetic alterations in oncogenic drivers, as detailed at the beginning of this review.

This heterogeneity is also reflected in the pattern of gene expression alterations documented for this tumor. From our analysis of the literature on ncRNAs, it is evident that many different small and long non‐coding genes contribute to the onset and progression of melanoma. These ncRNA alterations are reported as recurrent in several studies and in large cohorts, but the majority are is study‐dependent or not yet validated in large groups of patients. In addition, most of the published studies mixed primary and metastatic tumors, or did not discriminate between melanoma subtypes. For example, no analysis of the ncRNA profile of CSD and non‐CSD melanomas has been performed yet. We believe that this issue should be investigated in more detail in future studies.

As far as ncRNA research in melanoma is concerned, it is difficult to imagine that targeting a single miRNA or lncRNA could be an effective treatment for all melanoma patients. There is still much work to do in this field, especially *in vivo* studies for the validation of the most interesting miRNAs, lncRNAs or combination of these. Some interesting miRNA/lncRNA pairs which can boost tumor growth and dissemination, were identified. Despite this potential, the relationship and mutual interference between coding and non‐coding RNAs is still hardly studied because the quantification of all ncRNA types in the same sample is not usually available and computational analysis is complex.

We believe that this specific aspect of melanoma biology deserves further investigation and a proper integration with clinical and genomics data, in order to find all the missing pieces in the complex jigsaw puzzle of CM.

## Author contributions

All authors critically revised the literature, discussed the data, wrote and critically reviewed and revised this paper.

## Conflicts of interest

The authors have no conflicts of interest to declare.
